# Role of Intracoronary Imaging in Percutaneous Coronary Intervention for In-Stent Restenosis: A Prospective Observational Study

**DOI:** 10.14740/cr2224

**Published:** 2026-06-05

**Authors:** Ramya Das Nageri Kunnath, Pulugundla Varun, Sharath Nagesh, Harikrishnan Sivadasanpillai, Bijulal Sasidharan, Sreevilasam P. Abhilash

**Affiliations:** a Department of Cardiology, Sree Chitra Tirunal Institute for Medical Sciences and Technology, Thiruvananthapuram, Kerala, India

**Keywords:** In-stent restenosis, Intracoronary imaging, Intravascular ultrasound, Optical coherence tomography, Drug-eluting balloon, Drug-eluting stent

## Abstract

**Background:**

Despite advances in contemporary stent technology and procedural techniques, in-stent restenosis (ISR) remains a clinically significant cause of repeat revascularization. Coronary angiography, although the standard diagnostic modality, has limited ability to identify the underlying mechanism of ISR. Intravascular ultrasound (IVUS) and optical coherence tomography (OCT) may improve mechanism-based decision-making and procedural optimization. This study aimed to evaluate the impact of intracoronary imaging on ISR mechanism identification and treatment strategy during ISR percutaneous coronary intervention (PCI).

**Methods:**

This prospective observational study included 34 patients with 40 ISR lesions (60 previously implanted stents) undergoing IVUS- or OCT-guided PCI at a tertiary care center. Patients underwent detailed assessment of cardiovascular risk factors, angiographic classification of ISR, and systematic intracoronary imaging analysis. ISR lesions were classified as mechanically driven or tissue-proliferative based on imaging findings. The primary objective was to assess the impact of imaging on lesion preparation and definitive treatment strategy.

**Results:**

The mean age was 63 years, and 82.4% were male. Diffuse ISR was present in 57.5% of lesions. Intracoronary imaging identified mechanical and tissue-proliferative ISR in equal proportions (50% each). Mechanical ISR was more frequently associated with earlier presentation and structural complexity, including multisegment involvement and stent overlap zones. Intravascular imaging led to escalation of lesion modification strategies in 50% of lesions. Before imaging, drug-eluting balloon (DEB) therapy was planned in 55% and drug-eluting stent (DES) implantation in 15% of cases. Following imaging, DES implantation was performed in 50% of lesions, representing a significant shift in treatment strategy (McNemar test, P = 0.021).

**Conclusion:**

Both mechanical and tissue-proliferative mechanisms contributed substantially to ISR. Intracoronary imaging clarified the dominant ISR mechanism, guided lesion preparation, and modified definitive treatment strategy. These findings suggest a mechanism-based role for IVUS/OCT in lesion assessment and procedural planning during ISR PCI. Larger comparative studies with longer follow-up are required to determine whether imaging-guided ISR PCI improves clinical outcomes.

## Introduction

Percutaneous coronary intervention (PCI) has revolutionized the management of coronary artery disease, with advances in stent technology, guidewire design, and adjunctive devices improving procedural success and safety [1, 2]. Despite the advancements in PCI, in-stent restenosis (ISR) remains an important cause of recurrent ischemia and repeat revascularization, with contemporary data suggesting an annual clinical burden of 1–2% [3–5]. ISR PCI accounts for a substantial proportion of repeat coronary interventions in routine practice (5–10% of all PCIs) and may also present as acute coronary syndrome [6–10].

ISR is a heterogeneous process resulting from the interplay of mechanical and biological factors. Mechanical contributors include stent underexpansion, malapposition, stent fracture, and incomplete lesion coverage, whereas biological mechanisms include neointimal hyperplasia and neoatherosclerosis [11]. Patient-related factors such as diabetes mellitus, chronic kidney disease, obesity, dyslipidemia, hypertension, and smoking further increase the risk of restenosis. Lesion complexity, including calcification, bifurcation involvement, chronic total occlusion, and small-vessel disease, also contributes to ISR development [12–14].

Coronary angiography remains the standard diagnostic tool for ISR but has limited ability to define the underlying mechanism, vessel size, stent expansion, calcium burden, and neointimal characteristics. Intravascular ultrasound (IVUS) and optical coherence tomography (OCT) provide more detailed assessment of the stented segment and surrounding vessel, allowing mechanism-based evaluation and more precise procedural planning [3, 10]. Intracoronary imaging may influence lesion preparation, selection of drug-eluting balloon (DEB) versus repeat drug-eluting stent (DES) implantation, and post-procedural optimization. However, prospective real-world data linking imaging-defined ISR mechanisms with lesion preparation and treatment strategy remain limited [3, 10].

The present study aimed to evaluate the impact of intracoronary imaging in identifying ISR mechanisms and guiding procedural optimization during ISR PCI by correlating IVUS/OCT-defined lesion characteristics with lesion preparation and definitive treatment strategy.

## Materials and Methods

This was a prospective, observational study conducted at a tertiary care interventional cardiology unit. All patients undergoing PCI for ISR with available IVUS/OCT images were included in the study after taking written informed consent. STEMI requiring emergent PCI, ISR in coronary bypass grafts (saphenous vein graft (SVG)/left internal mammary artery (LIMA)), and severe vessel tortuosity preventing OCT/IVUS catheter advancement were excluded from the study. The study protocol was approved by the Institutional Ethics Committee of Sree Chitra Tirunal Institute for Medical Sciences and Technology before enrolment (Approval No. SCT/IEC/2473/NOVEMBER-2025), and was conducted in accordance with the ethical principles.

Baseline demographic data, cardiovascular risk factors, clinical presentation, and prior revascularization history were recorded. According to the interval from the index PCI, ISR was classified as early (< 30 days), late (30 days to 1 year), or very late (> 1 year), based on Society for Cardiovascular Angiography and Interventions (SCAI) definitions [10]. Diagnostic coronary angiography was performed using standard techniques. ISR was defined as luminal diameter stenosis ≥ 50% on coronary angiography accompanied by recurrent angina, objective evidence of myocardial ischemia, fractional flow reserve (FFR) < 0.80, IVUS-defined minimal luminal compromise, or diameter stenosis > 70% even in the absence of symptoms. Focal ISR was defined as restenosis ≤ 10 mm in length within the stented segment or at the stent edge. Diffuse ISR was defined as restenosis > 10 mm in length within the stented segment. Proliferative ISR was defined as restenosis > 10 mm extending beyond the stent margins, and occlusive ISR was defined as complete occlusion of the stented segment, according to the Mehran classification [15]. Tissue-proliferative ISR was defined as predominant neointimal tissue proliferation without a dominant mechanical abnormality. Neoatherosclerotic ISR was identified on intracoronary imaging by heterogeneous neointimal tissue, lipidic or calcific neointima, signal attenuation, microchannels, or thrombus, where applicable.

When more than one mechanism was present, the lesion was classified according to the dominant mechanism considered most relevant for treatment planning. Lesions with a major correctable mechanical abnormality, such as significant underexpansion, malapposition, or stent fracture, were classified as mechanically driven even when associated tissue proliferation was present. Lesions without a dominant mechanical abnormality but with predominant neointimal hyperplasia or neoatherosclerosis were classified as tissue-proliferative ISR.

The choice between IVUS and OCT was based on operator discretion, lesion morphology, catheter deliverability, renal function, need to minimize contrast use, and device availability. IVUS was more frequently used because of its wider availability, lower contrast requirement, and feasibility in tight or complex ISR lesions, whereas OCT was used when higher-resolution assessment of neointimal tissue morphology or neoatherosclerosis was required. If the imaging catheter could not cross the lesion, predilatation with a small compliant balloon (2.0–2.5 mm) was permitted to facilitate catheter passage.

IVUS analysis included minimal stent area (MSA), stent expansion, neointimal burden, calcium distribution, and reference vessel dimensions. OCT analysis included assessment of neoatherosclerosis, neointimal tissue characteristics, malapposition, calcium morphology, microchannels, and thrombus.

Based on intracoronary imaging findings, ISR lesions were classified as: 1) Mechanical ISR, defined by stent underexpansion, malapposition, stent fracture, or other dominant mechanical abnormalities. Stent underexpansion was defined as MSA < 80% of the average reference lumen area or absolute MSA < 5.0 mm^2^. 2) Tissue-proliferative ISR, defined by predominant neointimal tissue proliferation without a major mechanical abnormality, including neointimal hyperplasia or neoatherosclerosis with significant tissue burden [10, 16].

The pre-imaging treatment strategy was based on angiographic assessment and operator discretion rather than a fixed institutional protocol. Factors considered included ISR pattern and length, vessel size, suspected stent underexpansion, calcification, edge restenosis, number of prior stent layers, lesion location, and suitability for DEB versus repeat DES implantation. After intracoronary imaging, the final treatment strategy was modified according to the identified ISR mechanism, stent expansion, tissue burden, calcium distribution, and reference vessel dimensions.

**Figure 1:**
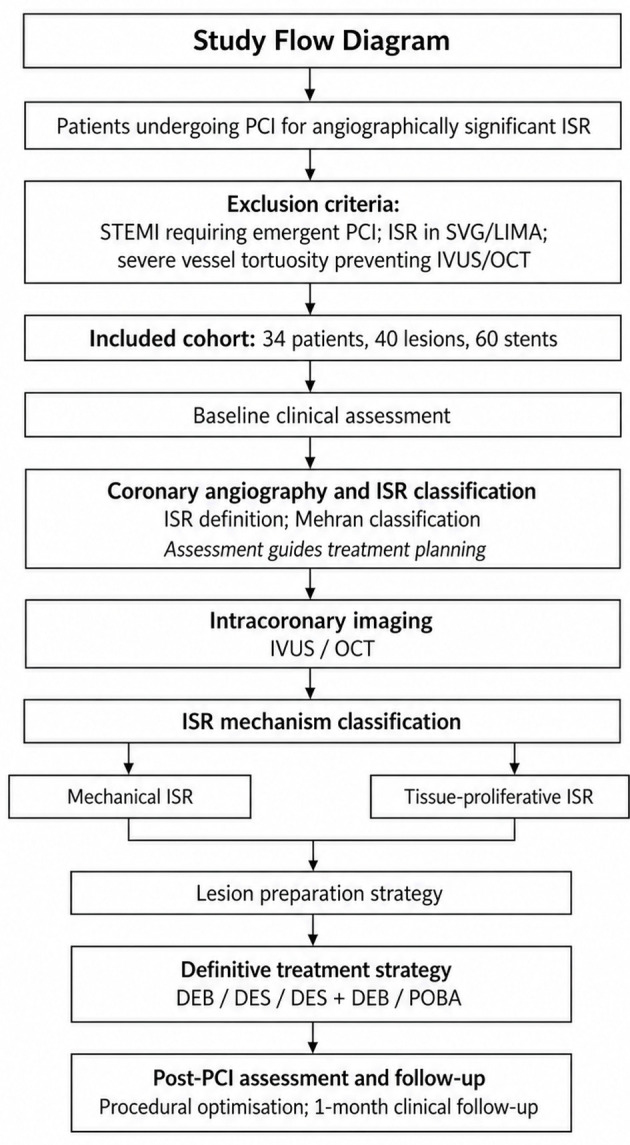
Study flow diagram. Flowchart showing patient selection, baseline clinical assessment, angiographic ISR classification, intracoronary imaging with IVUS/OCT, mechanism-based ISR classification, lesion preparation, definitive treatment strategy, and follow-up. ISR: in-stent restenosis; IVUS: intravascular ultrasound; OCT: optical coherence tomography.

Post-intervention angiography and, when feasible, repeat intravascular imaging were performed to assess final stent expansion, edge dissection, malapposition, coronary flow, and residual stenosis. Thrombolysis in myocardial infarction (TIMI) flow grade was documented. Serial electrocardiograms and cardiac troponin levels were obtained immediately after PCI and at 6 and 12 h after the procedure. The overall study design and procedural workflow are summarized in Figure 1.

### Statistical analysis

Continuous variables were expressed as mean ± standard deviation (SD) or median with interquartile range (IQR), and categorical variables as frequency and percentage. Chi-square or Fisher’s exact test was used for categorical variables, and the Mann-Whitney U test was used for continuous variables. The McNemar test was used to compare planned treatment strategy before imaging with final treatment strategy after imaging. A two-sided P value < 0.05 was considered statistically significant.

## Results

### Baseline characteristics

Between January 2025 and November 2025, 34 patients with 40 ISR lesions involving 60 previously implanted stents were included. The mean age was 63.06 ± 9.34 years, and 82.4% of patients were men. Chronic coronary syndrome was the most common presentation (52.9%), while 47.1% presented with acute coronary syndrome. Mean left ventricular ejection fraction was 51.56±11.09%. The median interval from index PCI to ISR presentation was 12.5 months (IQR 1–26.4). ISR was classified as early in 2.9%, late in 47.1%, and very late in 50.0% of cases.

At presentation, low-density lipoprotein (LDL) cholesterol was above target (> 55 mg/dL) in 58.8%, HbA1c was > 7.0% in 55.9%, and overweight/obesity (body mass index (BMI) > 23 kg/m^2^) was present in 82.4%. Active smoking at presentation was noted in 11.7%. Chronic kidney disease was present in 38.2%, and poor compliance to guideline-directed medical therapy was documented in 14.7%. Only two patients (5.9%) had optimal control of all major cardiovascular risk factors at presentation. Medication use at presentation was assessed from medical records. Five patients (14.7%) were not on antiplatelet therapy, four patients (11.8%) were on aspirin-based single antiplatelet therapy, 17 patients (50.0%) were on aspirin plus clopidogrel, and eight patients (23.5%) were on aspirin plus ticagrelor. High-intensity statin therapy was documented in 27 patients (79.4%), moderate-intensity statin therapy in two patients (5.9%), and five patients (14.7%) were not on statin therapy at presentation. Baseline characteristics are shown in Table 1. Clinical characteristics according to the dominant ISR mechanism are shown in Table 2.

**Table 1:** Baseline Characteristics

Variable	Overall cohort (N = 34)
Demographics	
Age (years), mean ± SD	63.1 ± 9.3
Male sex, n (%)	28 (82.4)
Female sex, n (%)	6 (17.6)
Cardiovascular risk factors	
Hypertension, n (%)	27 (79.4)
Diabetes mellitus, n (%)	24 (70.6)
Dyslipidemia, n (%)	28 (82.4)
Current or former smoker, n (%)	9 (26.5)
Obesity, n (%)	28 (82.4)
Uncontrolled cardiovascular risk factors^a^	
None (all risk factors controlled), n (%)	2 (5.9)
1 uncontrolled risk factor, n (%)	18 (52.9)
2 uncontrolled risk factors, n (%)	10 (29.4)
≥ 3 uncontrolled risk factors, n (%)	4 (11.8)
Other factors	
Chronic kidney disease, n (%)	13 (38.2)
Poor compliance to GDMT, n (%)	29 (85.3)
Clinical presentation	
Chronic coronary syndrome, n (%)	18 (52.9)
Acute coronary syndrome, n (%)	16 (47.1)
ST-elevation myocardial infarction (STEMI), n (%)	2 (5.9)
Non-ST-elevation myocardial infarction (NSTEMI), n (%)	9 (26.5)
Unstable angina (USA), n (%)	5 (14.7)
Baseline investigations	
Left ventricular ejection fraction, %	51.6 ± 11.1
LDL cholesterol, mg/dL	64.0 ± 23.4
HbA1c, %	7.6 ± 2.0
eGFR, mL/min/1.73 m^2^	71.4 ± 22.4
Time from index PCI to ISR	
Median delay, months (IQR)	12.5 (0.1–264)
Early ISR, n (%)	1 (2.9)
Late ISR, n (%)	16 (47.1)
Very late ISR, n (%)	17 (50.0)
Baseline antiplatelet therapy	
No antiplatelet therapy, n (%)	5 (14.7)
Aspirin alone, n (%)	4 (11.8)
Aspirin + clopidogrel, n (%)	17 (50.0)
Aspirin + ticagrelor, n (%)	8 (23.5)
Baseline statin therapy	
High-intensity statin, n (%)	27 (79.4)
Moderate-intensity statin, n (%)	2 (5.9)
No statin therapy, n (%)	5 (14.7)

Data are presented as mean ± SD, median (IQR), or number (percentage), as appropriate. ^a^Uncontrolled cardiovascular risk factors were defined as LDL cholesterol ≥ 55 mg/dL, HbA1c ≥ 7.0%, or body mass index ≥ 23 kg/m^2^ at presentation. eGFR: estimated glomerular filtration rate; GDMT: guideline-directed medical therapy; HbA1c: glycated hemoglobin; IQR: interquartile range; LDL: low-density lipoprotein; ISR: in-stent restenosis; PCI: percutaneous coronary intervention; SD: standard deviation.

**Table 2:** Clinical Characteristics by ISR Mechanism

Variable	Mechanical ISR (n = 17)	Tissue-proliferative ISR (n = 17)	P value
Age (years), median (IQR)	67 (58.5–73)	62 (56.5–70)	0.085
BMI (kg/m^2^), median (IQR)	26.0 (24.08–28.51)	24.8 (23.5–26.5)	0.17
eGFR (mL/min/1.73 m^2^), median (IQR)	64.0 (49.0–88.0)	69.0 (52-98.5)	0.760
LDL (mg/dL), median (IQR)	64.0 (49-83)	56.0 (47.5–68.5)	0.394
HbA1c (%), median (IQR)	8 (5.95–8.88)	7 (6.00–8.58)	0.708
LVEF (%), median (IQR)	48 (42.5–57.5)	51.0 (40.5–66)	0.518
Acute coronary syndrome, n (%)	10 (56.6)	8 (44.4)	0.732
Chronic coronary syndrome, n (%)	7 (43.8)	9 (56.3)	
ISR timing	Mechanical ISR (n = 20)	Tissue-proliferative ISR (n = 20)	
ISR within 1 year, n (%)	16 (80.0)	4 (20.0)	< 0.001*
Very late ISR, n (%)	4 (20.0)	16 (80.0)	

*Statistically significant. BMI: body mass index; eGFR: estimated glomerular filtration rate; HbA1c: glycated hemoglobin; IQR: interquartile range; LDL: low-density lipoprotein; LVEF: left ventricular ejection fraction; ISR: in-stent restenosis.

### Angiographic characteristics

Forty ISR lesions were treated with intravascular imaging guidance. The left anterior descending artery was the most frequently involved vessel (52.5%), followed by the right coronary artery (22.5%), left circumflex artery (15.0%), and left main bifurcation (10.0%). Progressive atherosclerotic disease in non-target vessels was assessed angiographically and was observed in 16 patients (47.1%). Among these, 11 patients had ISR in additional previously stented vessels and three patients had progression in previously non-stented segments. Intravascular imaging was performed for the target ISR lesion undergoing intervention and was not routinely performed for non-target vessels unless clinically required.

**Figure 2:**
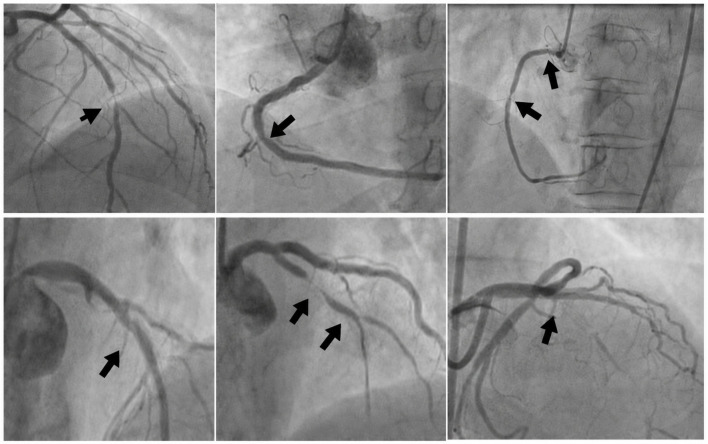
Angiographic images from study patients demonstrating patterns of ISR according to the Mehran classification. Upper panel (left to right) shows focal ISR patterns: Mehran IB, Mehran IC, and Mehran ID. Lower panel (left to right) illustrates diffuse ISR patterns: pattern II (diffuse intrastent), pattern III (diffuse proliferative), and pattern IV (total occlusion). Black solid arrows indicate the site and extent of ISR within or adjacent to the stented segment. ISR: in-stent restenosis.

**Figure 3:**
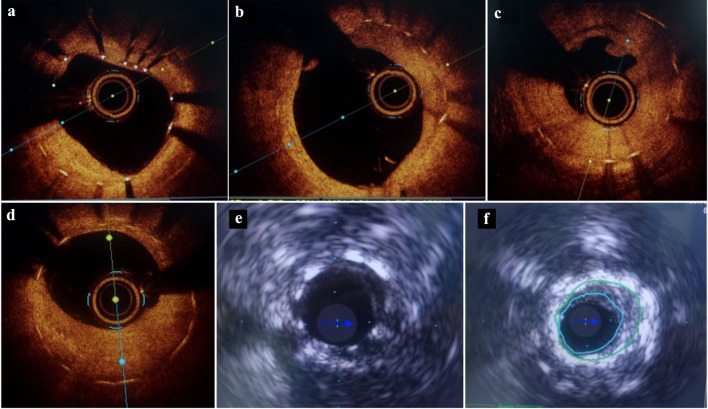
Representative OCT and IVUS images demonstrating mechanisms of ISR. Panels (a)–(d) show optical coherence tomography cross-sectional images of ISR. Panel (a) demonstrates eccentric neointimal hyperplasia within the stented segment causing significant luminal compromise with well-apposed stent struts. Panel (b) shows diffuse, predominantly homogeneous neointimal proliferation resulting in concentric luminal narrowing. Panel (c) demonstrates heterogeneous neointimal tissue with signal attenuation suggestive of complex tissue composition, consistent with neoatherosclerosis. Panel (d) shows concentric neointimal hyperplasia with significant tissue burden leading to luminal narrowing. Panels (e) and (f) show intravascular ultrasound images. Panel (e) demonstrates significant neointimal tissue proliferation within the stented segment without an obvious mechanical abnormality. Panel (f) demonstrates stent underexpansion with reduced minimal stent area and persistent luminal compromise, consistent with mechanically driven ISR. ISR: in-stent restenosis; IVUS: intravascular ultrasound; OCT: optical coherence tomography.

Details of previously implanted stents were collected wherever available. Among the 40 ISR lesions, second-generation DES were involved in 36 lesions (90.0%), first-generation DES in three lesions (7.5%), and bare-metal stents in one lesion (2.5%). At the stent level, 60 previously implanted stents were analyzed. Lesion and stent characteristics according to the dominant ISR mechanism are summarized in Table 3. However, the specific stent manufacturer or brand was not consistently available from medical records or prior PCI documentation. Because of the very small sample size, predominance of second-generation DES, and incomplete manufacturer/brand-level data, subgroup analysis according to stent type, manufacturer, or brand was not performed. Diffuse ISR was present in 57.5% of lesions, while focal ISR was present in 42.5%. Mehran class III ISR accounted for 32.5% of lesions. Angiographic patterns are shown in Figure 2 and representative OCT and IVUS images illustrating tissue-proliferative and mechanically driven ISR are shown in Figure 3.

**Table 3:** Lesion Characteristics by ISR Mechanism

Variable	Mechanical ISR (n = 20)	Tissue- proliferative ISR (n = 20)	P value
Target vessel			
Left anterior descending artery, n (%)	7 (35.0)	14 (70.0)	0.172
Left circumflex artery, n (%)	4 (20.0)	2 (10.0)	
Right coronary artery, n (%)	6 (30.0)	3 (15.0)	
Left main bifurcation, n (%)	3 (15.0)	1 (5.0)	
Stent characteristics			
Total stent length (mm), median (IQR)	42.0 (37.0–63.0)	31.0 (25.0–39.5)	0.002*
Minimum stent diameter, (mm)	3.00 (2.50–3.00)	3.00 (2.75–3.38)	0.542*
Overlap involved, n (%)	13 (65.0)	5 (25.0)	0.011*
Number of ISR segments			
Single segment, n (%)	8 (40.0)	16 (80.0)	0.010*
≥ 2 segments, n (%)	12 (60.0)	4 (20.0)	
Minimum stent diameter (mm), median (IQR)	3.00 (2.50–3.00)	3.00 (2.75–3.38)	
Tissue burden (% area), median (IQR)	65 (45–76)	66 (58–78)	0.176
Pre-intervention minimum stent area (mm^2^), median (IQR)	5.32 (3.35–6.35)	5.20 (4.58–5.60)	0.231
Pre-intervention minimum luminal area (mm^2^), median (IQR)	1.98 (1.42–2.22)	1.84 (1.29–3.13)	0.835
Pre-stent expansion (%), median (IQR)	64.6 (44.8–78.6)	78.3 (56.6–83.0)	0.04

*Statistically significant. IQR: interquartile range; ISR: in-stent restenosis.

### Intracoronary imaging–based lesion characteristics

Intravascular imaging was performed with IVUS in 33 lesions (82.5%) and OCT in seven lesions (17.5%). Balloon predilatation before imaging was required in 30% of lesions to allow catheter passage. Mechanical and tissue-proliferative ISR accounted for 50% of lesions each.

Mechanical ISR was associated with earlier presentation, predominantly within 12 months of the index PCI, whereas tissue-proliferative ISR was more common in later presentations (P < 0.001). There was no significant difference in target vessel distribution between the two groups. Mechanical ISR was associated with greater structural complexity, including longer total stented length, more frequent multisegment involvement, and more frequent overlap zones. Calcification was significantly more common in mechanically driven ISR (70% vs. 25%, P = 0.004), whereas heterogeneous neointimal morphology was more commonly observed in tissue-proliferative ISR (80% vs. 50%, P = 0.047). Imaging-based lesion characteristics according to ISR mechanism are summarized in Table 4.

**Table 4:** Imaging-Based Lesion Characteristics by ISR Mechanism

Variable	Mechanical ISR (n = 20)	Tissue-proliferative ISR (n = 20)	P value
Imaging modality			0.677
IVUS, n (%)	17 (85.0%)	16 (80.0%)	
OCT, n (%)	3 (15.0%)	4 (20.0%)	
Mechanical abnormality			
Underexpansion, n (%)	12 (60.0%)	6 (30.0%)	-
Malapposition, n (%)	3 (15.0%)	0	
Stent fracture, n (%)	1 (5.0%)	0	
Neointimal tissue pattern			0.027*
Homogeneous, n (%)	10 (50.0%)	4 (20.0%)	
Heterogeneous, n (%)	8 (40.0%)	16 (80.0%)	
Thrombus, n (%)	2 (10.0%)	0	
Calcification			0.004*
Present, n (%)	14 (70.0%)	5 (25.0%)	
Absent, n (%)	6 (30.0%)	15 (75.0%)	

*Statistically significant. ISR: in-stent restenosis; IVUS: intravascular ultrasound; OCT: optical coherence tomography.

### Lesion preparation and treatment optimization

Intracoronary imaging led to escalation of lesion modification strategy in 50% of lesions (20/40) compared with the plan based on angiography alone. This rate was similar in mechanically driven and tissue-proliferative ISR (50% in both groups, P = 0.527).

**Figure 4:**
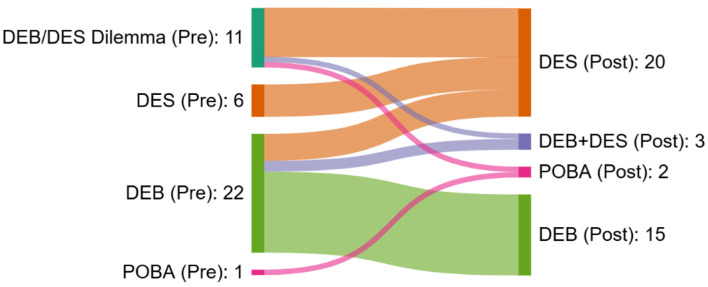
Imaging-guided change in treatment strategy for ISR. Pre-imaging strategies included DEB (55%), DES (15%), DES with possible DEB conversion (27.5%), and POBA (2.5%). Following intracoronary imaging, final strategies were DES (50%), DES + DEB (7.5%), and POBA (5%). The Sankey diagram illustrates reclassification of treatment strategy after imaging, predominantly toward stent-based approaches. DEB: drug-eluting balloon; DES: drug-eluting stent; ISR: in-stent restenosis; POBA: plain old balloon angioplasty.

Before imaging, a DEB-based strategy had been planned in 55% of lesions, DES in 15%, DES with possible DEB conversion in 27.5%, and plain old balloon angioplasty (POBA) in 2.5%. After imaging, the final treatment strategy changed substantially: DES was used in 50% of lesions, DES + DEB in 7.5%, and POBA alone in 5%. Overall, imaging produced a significant shift in definitive treatment strategy, with increased use of stent-based treatment after mechanism clarification (McNemar test, P = 0.021) (Fig. 4). The DES + DEB strategy was used in selected complex or multisegment ISR lesions where focal segments with mechanical failure, edge disease, or major residual stenosis required repeat stenting, while adjacent restenotic segments with predominant tissue proliferation were treated with DEB to avoid unnecessary additional metal layers.

**Figure 5:**
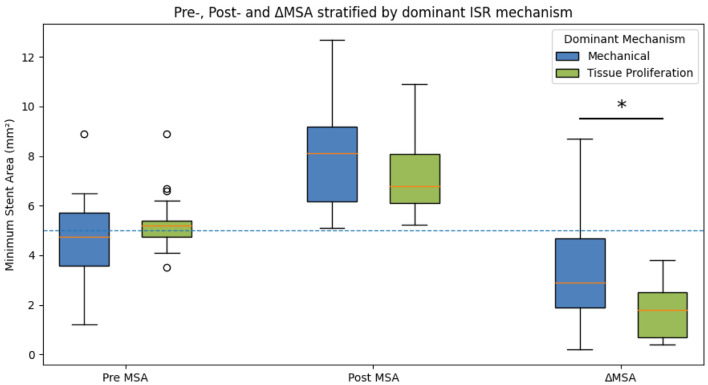
Pre-, post- and ΔMSA stratified by ISR mechanism. Box-and-whisker plots depicting pre-intervention minimum stent area (pre-MSA), post-intervention minimum stent area (post-MSA), and change in minimum stent area (ΔMSA) stratified by ISR mechanism (mechanically driven vs. tissue-proliferative ISR). Boxes represent the interquartile range (25th–75th percentile), the central line denotes the median, and whiskers indicate the range excluding outliers. The dashed horizontal line represents a reference MSA threshold of 5.0 mm^2^. ISR: in-stent restenosis.

Imaging-guided post-dilatation for optimization was required in 52% of lesions (21/40; 95% confidence interval (CI) 36.5–67.5%). Final post-procedural MSA was comparable between the two ISR groups. However, the absolute gain in lumen area (ΔMSA) was significantly greater in mechanically driven ISR than in tissue-proliferative ISR (P = 0.010) (Fig. 5). Treatment optimization data are presented in Table 5.

**Table 5:** Treatment and Optimization Characteristics by Dominant ISR Mechanism

Variable	Mechanical ISR (n = 20)	Tissue-proliferative ISR (n = 20)	P value
Lesion preparation			0.54
NC balloon use, n (%)	12 (30)	12 (30)	
Cutting balloon, n (%)	6 (15)	7 (17.5)	
Scoring balloon, n (%)	0 (0%)	1 (2.5%)	
OPN balloon, n (%)	1 (2.5%)	0 (0%)	
Atherectomy/IVL, n (%)	1 (2.5%)	0 (0%)	
Treatment strategy			0.45
POBA, n (%)	2 (5)	0 (0)	
DEB only, n (%)	7 (17.5)	8 (20)	
DES, n (%)	9 (22.5)	11 (27.5)	
DEB + DES, n (%)	2 (5)	1 (2.5)	
Post-intervention minimum stent area (mm^2^), median (IQR)	8.10 (5.80–8.45)	6.80 (5.90–8.44)	
Post-stent expansion (%), median (IQR)	99.0 (90.0–110.0)	95.0 (89.0–107.8)	
Δ Minimum stent area (mm^2^), median (IQR)	2.88 (1.75–4.20)	1.80 (0.63–2.70)	

DEB: drug-eluting balloon; DES: drug-eluting stent; IQR: interquartile range; ISR: in-stent restenosis; IVL: intravascular lithotripsy; NC: non-compliant; OPN: high-pressure; POBA: plain old balloon angioplasty.

### Procedural and early outcomes

Immediate procedural success was achieved in 94.3% of patients. Two patients had residual underexpansion despite optimization attempts. Periprocedural myocardial injury, defined as troponin elevation > 5 times the upper limit of normal, occurred in four patients (11.4%), none of whom had ischemic symptoms or new electrocardiographic changes.

At 1-month follow-up, no patient experienced major adverse cardiac events. Most patients (94.3%) were symptom free and in NYHA functional class I. Over a median follow-up of 4 months, target vessel failure occurred in one patient (2.94%).

## Discussion

ISR remains a relevant clinical problem despite advances in stent platforms, pharmacotherapy, and procedural techniques. Previous imaging and pathological studies have shown that ISR is not a uniform entity but includes a spectrum of mechanisms ranging from stent underexpansion, malapposition, and fracture to neointimal hyperplasia and neoatherosclerosis [1–4, 17–20]. Contemporary consensus documents recommend intracoronary imaging when the mechanism of ISR is uncertain, particularly when repeat stenting or advanced lesion preparation is being considered [2, 3, 10]. Prior real-world OCT-guided PCI data, including the LightLab Initiative, have also shown that imaging can modify operator strategy more frequently in ISR than in *de novo* lesions [21]. The present study adds to this literature by prospectively describing the relationship between imaging-defined ISR mechanisms and procedural decision-making in a tertiary care setting.

### Clinical profile and presentation of ISR

The study cohort demonstrated a high burden of cardiovascular risk factors, including diabetes mellitus, hypertension, dyslipidemia, obesity, and chronic kidney disease, with suboptimal control of one or more risk factors in the majority of patients. This profile is consistent with prior observational studies and registry data, which have shown that metabolic and inflammatory risk states contribute to adverse vascular healing and restenosis [5–7, 12]. A substantial proportion (47%) of patients presented with acute coronary syndromes, supporting previous observations that ISR is not exclusively a stable clinical entity and may present with clinically significant ischemia, particularly in late and very late phases [5, 6].

### Timing of ISR and mechanistic substrate

A key finding of this study was the association between the timing of ISR presentation and the dominant mechanism identified by intravascular imaging. Mechanically driven ISR predominated in lesions presenting within the first year after index PCI, whereas tissue-proliferative ISR was more frequent in very late presentations. This temporal distribution aligns with established pathophysiological frameworks described in prior clinical, imaging, and pathological studies, in which early ISR is commonly associated with stent underexpansion, malapposition, or structural deformation, while progressive neointimal proliferation and neointimal atherosclerosis become more prevalent over time [1–4].

### Structural complexity and mechanical ISR

Mechanically driven ISR was associated with longer total stented length, involvement of stent overlap zones, and multisegment disease. These findings are consistent with prior intravascular imaging studies demonstrating that increased metal burden and overlap regions are susceptible to impaired expansion and altered vessel biomechanics [9–11]. Calcification was more prevalent in mechanically driven ISR, supporting the established role of calcific resistance in limiting optimal stent expansion and contributing to early restenosis [10]. These structural characteristics are often underestimated by angiography, underscoring the value of intravascular imaging in identifying correctable mechanical constraints during ISR PCI.

### Tissue proliferation and neointimal characteristics

Tissue-proliferative ISR constituted a substantial proportion of lesions, particularly in very late presentations and was characterized by heterogeneous neointimal tissue patterns on intravascular imaging. Prior OCT and IVUS studies have demonstrated that heterogeneous or layered neointimal patterns reflect complex tissue composition and, in some cases, neoatherosclerosis [17–20]. Such tissue characteristics are not reliably distinguished by angiography but have important therapeutic implications, particularly in selecting balloon-based strategies versus repeat stenting. The present findings reinforce that intravascular imaging contributes not only to the identification of mechanical failure but also to the characterization of neointimal tissue, which directly informs lesion preparation and treatment strategy.

### Impact of intravascular imaging on decision-making

The principal objective of this study was to assess the procedural impact of intravascular imaging in ISR PCI. Imaging influenced multiple stages of intervention, including identifying the mechanism, escalation of lesion-preparation strategies, modification of definitive treatment selection, and objective assessment of procedural optimization. These observations are consistent with prior pooled analyses and real-world OCT-guided PCI data demonstrating that intravascular imaging frequently alters operator decision-making compared with angiography alone [21–23]. Notably, the LightLab Initiative demonstrated that OCT-guided PCI resulted in a higher rate of strategy modification in ISR compared with *de novo* lesions, highlighting the particular value of imaging in this setting [21]. The present study extends these observations by demonstrating that imaging impacts the entire ISR PCI workflow rather than a single procedural step.

### PCI optimization and procedural efficacy

Although the final post-intervention minimum stent area was similar between ISR groups, mechanically driven ISR demonstrated greater absolute lumen gain following imaging-guided intervention. This finding is consistent with prior imaging-guided PCI studies showing that correction of mechanical constraints yields significant improvements in stent expansion, whereas biologically mediated restenosis may demonstrate more modest acute luminal gains despite adequate optimization [24–26]. Importantly, intravascular imaging enabled acceptable final expansion and apposition in both ISR phenotypes.

### Limitations

This was a single-center observational study with a very small sample size and very short clinical follow-up. Therefore, the study was not powered to evaluate long-term clinical outcomes or establish causal associations between intracoronary imaging and clinical events. The absence of a control group limits any inference regarding the clinical superiority of imaging-guided ISR PCI over angiography-guided treatment. The very short 1-month follow-up is another major limitation, as it does not allow assessment of recurrent restenosis, late target lesion failure, or long-term major adverse cardiac events. Imaging modality selection and treatment strategy were based on operator discretion, reflecting real-world practice but introducing potential selection bias. Because ISR mechanisms frequently overlap, classification into two dominant categories may oversimplify the biological and mechanical continuum of ISR. Mixed-mechanism lesions were categorized according to the dominant mechanism considered most relevant to treatment planning. Although baseline antiplatelet and statin therapy were recorded, the study was not powered to assess the independent effect of medication adherence or individual pharmacological regimens on ISR mechanism or procedural outcomes.

### Conclusion

**Figure 6:**
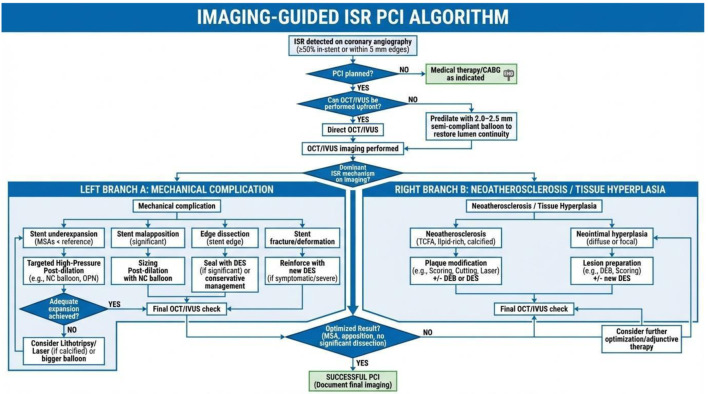
Imaging-guided decision aid algorithm for the management of in-stent restenosis.

In this prospective observational study, both mechanical and tissue-proliferative mechanisms contributed substantially to ISR. Intracoronary imaging clarified the dominant ISR mechanism, guided lesion preparation, and modified treatment selection during ISR PCI. These findings suggest that IVUS/OCT is useful in lesion assessment and treatment planning during ISR PCI. However, given the observational design, absence of a control group, very small sample size, and short follow-up, these findings should be interpreted as procedural and hypothesis-generating. Larger comparative studies with longer follow-up are required to determine whether imaging-guided ISR PCI improves long-term clinical outcomes. The proposed imaging-guided ISR PCI algorithm is given in Figure 6.

## Data Availability

The data supporting the findings of this study are available from the corresponding author upon reasonable request.
